# Quantifying promoter‐specific *Insulin‐like Growth Factor 1* gene expression by interrogating public databases

**DOI:** 10.14814/phy2.13970

**Published:** 2019-01-03

**Authors:** Peter Rotwein

**Affiliations:** ^1^ Department of Biomedical Sciences Paul L. Foster School of Medicine Texas Tech Health University Health Sciences Center El Paso Texas

**Keywords:** Gene expression, gene regulation, genomics, IGF1

## Abstract

The actions of insulin‐like growth factor 1 (IGF1), a small, secreted protein, are essential for normal somatic growth in children and are important for tissue regeneration and repair in adults. Similar functions are conserved in other mammalian species. *IGF1* gene regulation is complicated in mammals, with transcription being controlled by different hormonal, nutritional, and tissue‐specific inputs. Quantifying *IGF1* gene expression in different organs and tissues also has been difficult because of the variable contributions of its two promoters and because of the lack of standard platforms for analysis. Here, I have taken advantage of the wealth of information found in publicly accessible RNA‐sequencing libraries to measure steady‐state levels of *IGF1* mRNAs from human and macaque, species chosen because they are not readily tractable experimental organisms, yet retain similar *IGF1* gene organization. Results demonstrate that *IGF1* transcripts are highly expressed in fat and liver in both species, and are induced during human adipocyte differentiation. *IGF1* mRNAs also are increased in macaque skeletal muscle after selected dietary manipulations. In the organs and tissues examined, *IGF1* promoter 1 appears to be far more active than promoter 2. Collectively, these observations show that interrogating large‐scale public genomic resources is an effective strategy for quantifying gene expression across different tissues and species.

## Introduction

The conserved peptide, insulin‐like growth factor 1 (IGF1) is a 70‐amino acid, single‐chain secreted protein that plays a key role in many physiological and pathophysiological processes in humans and other mammals (Le Roith et al. 2001; LeRoith 2008; Gallagher and LeRoith 2011). IGF1 is essential for normal pre‐ and postnatal growth in human children and in other mammalian species (Powell‐Braxton et al. 1993; Woods et al. 1996; Le Roith et al. 2001; Lupu et al. 2001; LeRoith 2008), and also is involved in regulating aspects of intermediary metabolism, tissue regeneration, and disease pathogenesis in adults (Berryman et al. 2008; Gallagher and LeRoith 2011; Pollak 2012; Gems and Partridge 2013).

Recent advances in genomics and genetics now provide unprecedented opportunities for understanding comparative physiology and evolution, and for gaining insights into potentially conserved regulatory processes among different species (Acuna‐Hidalgo et al. 2016; Katsanis 2016; Quintana‐Murci 2016) through analysis of data deposited in public genomic and gene expression databases (Manolio et al. 2017). For example, studies of the *IGF1*/*Igf1* gene and locus have revealed similarities among 25 different mammals in terms of exon‐intron organization, the presence of tandem promoters, each with a distinct leader exon, and conservation of protein‐coding regions, but have identified substantial differences among putative growth hormone (GH)‐activated transcriptional enhancers (Rotwein 2018a). In contrast, in 21 nonmammalian vertebrates, the *IGF1* gene contains a single‐gene promoter and conserved coding exons, lacks the GH‐regulated enhancers found in mammals, and encodes “extra” exons in birds, reptiles, and fish (Rotwein 2018c). Analogous diversity has been discovered among mammalian and nonmammalian *IGF2*/*Igf2* genes (Rotwein 2018b,d), with the added feature that both *Igf2* and its complex multi‐gene locus are far simpler in nonmammalian vertebrates than in mammals (Rotwein 2018b).

Despite these examples, the full potential of the resources found in publically accessible genomic repositories to drive biomedical research questions has not been realized yet. Large amounts of untapped gene expression data are present in the Sequence Read Archive of the National Center for Biotechnology Information (SRA NCBI), a searchable database that as of December 8, 2018 held 8,706,196,291,902,060 nucleotides of open‐access information from many different species. These data have been deposited by investigators using a variety of ‘next‐generation’ DNA‐sequencing platforms to generate computer files each containing up to billions of nucleotides of searchable RNA‐sequencing results from different organisms, organs and tissues, developmental stages, and experimental models. Here I have used this information to assess promoter‐specific expression of *IGF1* transcripts in different organs and tissues from human and macaque, species chosen because they are not readily tractable experimental organisms, but have similar *IGF1* gene structures and DNA sequences. Results show that *IGF1* transcripts are highly expressed in fat and liver in both species, and are induced during human adipocyte differentiation. *IGF1* mRNAs also are increased in macaque skeletal muscle with different dietary manipulations. In the organs, tissues and experimental models examined, *IGF1* promoter 1 appears to be far more active than promoter 2. These results illustrate how large‐scale shared genomic resources can empower an individual investigator to explore new experimental paradigms, and define an effective approach to generate gene expression data that is easily the equal of quantitative RT‐PCR or similar laboratory‐based strategies.

## Materials and Methods

### Databases and analyses

Information on *IGF1* genes was obtained from the Ensemble (www.ensemble.org) and UCSC Genome Browsers (https://genome.ucsc.edu), using human genome assembly, GRCh38.p12, and macaque genome assembly, Mmul_8.0.1. The Genotype‐Tissue Expression project (GTEx) portal (https://www.gtexportal.org/) was a source of data on *IGF1* gene expression in different human tissues. RNA‐sequencing information was extracted from the Sequence Read Archive of the National Center for Biotechnology Information (SRA NCBI; www.ncbi.nlm.nih.gov/sra) by querying the datasets listed in Tables 2 and 3 with the 60‐base pair (bp) DNA probes found in Table 1. Searches were performed using the megablast option optimized for highly similar sequences; maximum target sequences of 10000 (this parameter may be set from 50 to 20,000); expect threshold of 10; word size of 11; match/mismatch scores of 2, −3; gap costs of existence 5, extension 2; and low‐complexity regions filtered.

**Table 1:** Probes for screening RNA‐sequencing libraries

Species	Gene	Probe
Human	*IGF1* exons 1–3	TTTAAGTGCTGCTTTTGTGATTTCTTGAAGGTGAAGATGCACACCATGTCCTCCTCGCAT
	*IGF1* exons 2–3	CTGGAACAAACAAAAATGATTACACCTACAGTGAAGATGCACACCATGTCCTCCTCGCAT
	*MRPS17* exon 3	TATTTTAATAAGCGGAAAACCTACTTTGCTCACGATGCCCTTCAGCAGTGCACAGTTGGG
	*FABP4* exon 2	GAGTGGGCTTTGCCACCAGGAAAGTGGCTGGCATGGCCAAACCTAACATGATCATCAGTG
Macaque	*IGF1* exons 1‐3	TTTAAGTGCTGCTTTTGTGATTTCTTGAAGGTGAAGATGCACACCATGTCCTCCTCGCAT
	*IGF1* exons 2–3	CTGGAACAAACAAAAATGATTACACCTACAGTGAAGATGCACACCATGTCCTCCTCGCAT
	*MRPS17* exon 3	TATTTTAATAAGCGGAAAACCTACTTTGCGCACGATGCCCTTCAGCAGTGCACAGTTGGG

## Results

### *IGF1* genes and gene expression in mammals

**Figure 1:**
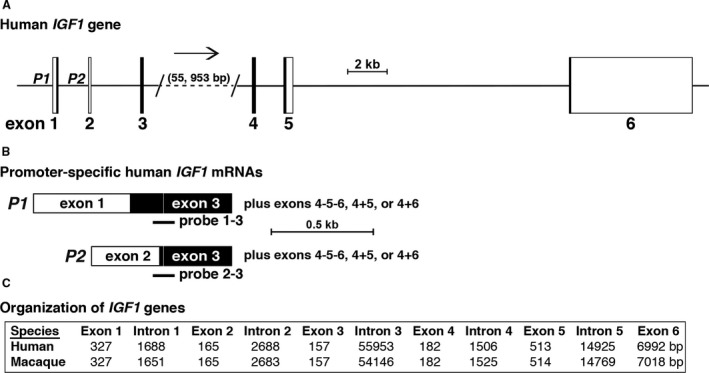
Organization of the human and macaque *IGF1* genes. (A) Schematic of the human *IGF1* gene. Exons are depicted as boxes, with coding regions in black and noncoding DNA in white, and introns as horizontal lines. Angled parallel lines indicate discontinuities, with the distance being spanned listed in parentheses. *P1* and *P2* indicate the locations of promoters 1 and 2, respectively. A scale bar is shown. (B) Diagram of the two classes of promoter‐specific human *IGF1* mRNAs, which are transcribed by *P1* or *P2*, and include exon 1 or exon 2, respectively. Specific probes for searching SRA‐NCBI libraries are indicated. (C) Comparison of *IGF1* genes from human and macaque, showing the lengths of all exons and introns.

*IGF1* is a 6‐exon, 5‐intron gene in humans, macaque, and in many other mammals (Rotwein 2017, 2018a) (Fig. 1). The vast majority of mammalian *IGF1* genes that have been examined to date appear to have two promoters, each with it own unique leader exon (Rotwein 2017, 2018a) (Fig. 1A), although in very few examples have the promoters been assessed functionally (Hall et al. 1992; Mittanck et al. 1997; Wang et al. 1998, 2000; Varco‐Merth and Rotwein 2014). The fact that each promoter‐specific leader exon (exons 1 or 2) splices into common exon 3 (Fig. 1B) leads to a strategy in which *IGF1* transcripts directed by each promoter can be analyzed individually, and both quantified and calibrated against each other (Fig. 1B, Table 1).

### *IGF1* gene expression in human tissues

**Figure 2:**
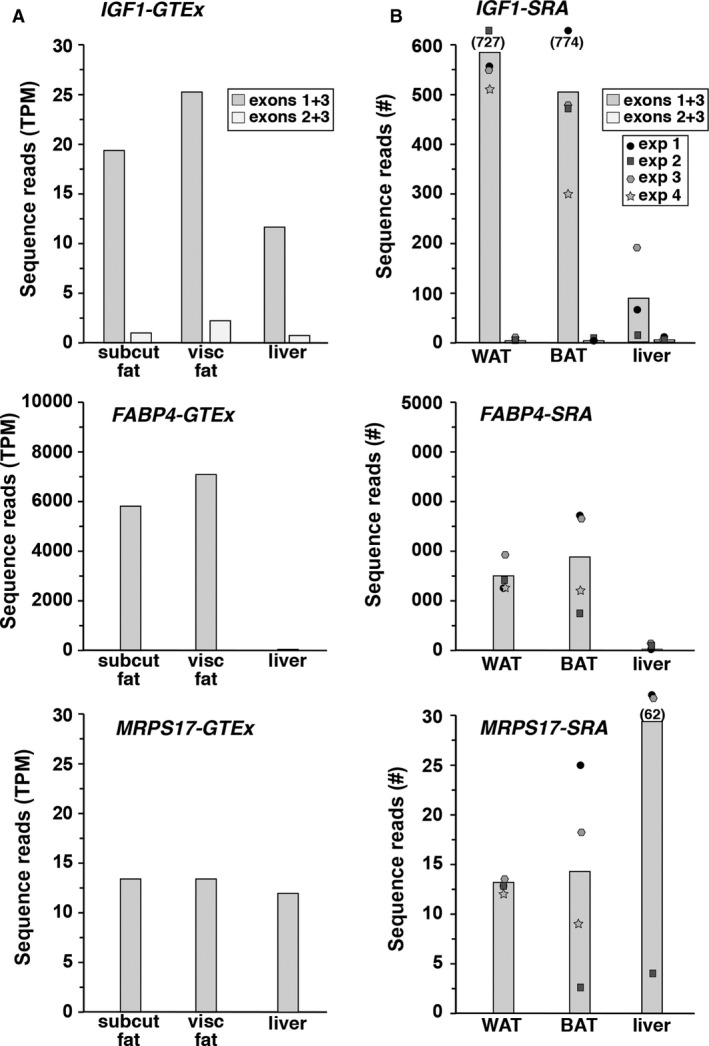
*IGF1* gene expression in human organs and tissues. (A) Transcript levels for *IGF1* (exons 1 + 3 = directed by *P1*; exons 2 + 3 = directed by *P2*), *FABP4*, and *MRPS17* (top to bottom panels, respectively) were measured in subcutaneous (subcut) and visceral (visc) fat, and liver by examining GTEx. Mean aggregate data are plotted for RNA from 442, 355, and 175 individuals, respectively, for subcutaneous fat, visceral fat, and liver (TPM = transcripts per kilobase million reads). (B) Transcript levels for *IGF1* (exons 1 + 3 = directed by *P1*; exons 2 + 3 = directed by *P2*), *FABP4*, and *MRPS17* (top to bottom panels, respectively) were obtained from white and brown fat (WAT and BAT, respectively), and liver by interrogating the gene expression libraries listed in Table 2. Mean results of 3–4 experiments are shown in the bar graphs, with individual data being plotted as circles, squares, or stars.

Most studies on *IGF1* gene regulation have used animal models, principally mice and rats, with efforts being focused on transcriptional control by GH or by nutritional factors (Adamo et al. 1991; Hall et al. 1992; Adamo 1995), and have found that highest levels of *IGF1* mRNA in these species were detected in liver (Hall et al. 1992; Gosteli‐Peter et al. 1994; Adamo 1995; Waxman and O'Connor 2006). The recent release of data from GTEx (GTEx consortium, 2015; Battle et al. 2017) now provides an opportunity to assess otherwise unavailable human samples. At present, this resource has global gene expression data from more than 50 different human organs and tissues aggregated from several hundred individuals. Initial evaluation of this information revealed that *IGF1* mRNA is expressed in 34 different human tissues and organs, but at steady‐state values that varied over a ~25‐fold range (from ~1.2 to >29 transcripts per kilobase million reads (TPM, data not shown), with the highest levels of expression found in fat depots (Fig. 2A), and in mammary tissue (data not shown), which consists principally of adipose cells (Neville 1999). Remarkably, the abundance of *IGF1* mRNA in human liver was 50–60% of what was measured in visceral or subcutaneous fat (Fig. 2A), although in all three samples, levels of *MRPS17*, a presumptively constitutively expressed gene, appeared to be relatively constant, and the fat‐differentiation‐specific mRNA, *FABP4*, which encodes a lipid binding protein (Hotamisligil and Bernlohr 2015), was appropriately highly abundant in fat (Fig. 2A). Of note, *IGF1* promoter 1 appeared to direct the vast majority of *IGF1* transcripts in the GTEx cohort (Fig. 2A).

To expand on these observations, *IGF1* transcripts directed by promoters 1 and 2 were examined by interrogating RNA‐sequencing libraries from the SRA NCBI resource (Table 2), using two 60‐bp DNA probes that spanned the junctions of *IGF1* exons 1 and 3, and exons 2 and 3, respectively (Fig. 1B, Table 1). Queries of several libraries (Table 2) with each of the probes demonstrated that mRNAs directed by both *IGF1* promoters are present in human liver, and in both white and brown fat, with mRNA levels being five to six times higher in the latter than in the former (Fig. 2B). In all samples, promoter 1‐derived transcripts were far more abundant than those regulated by promoter 2 (Fig. 2B). In addition, as observed in the GTEx results, *FABP4* mRNA levels were far higher in fat than in liver (Fig. 2B), although the control mRNA, *MRPS17*, was four times more abundant in liver (Fig. 2B). Taken together, the results in Figure 2 show that information from GTEX and SRA NCBI match up fairly well with regard to general patterns of *IGF1* gene expression in different human organs and tissues. In both data resources, the highest levels of *IGF1* mRNA were found in fat (Fig. 2).

**Table 2:** Human tissue RNA‐sequencing libraries screened for gene expression

	Experiment	Platform	Layout	Bases sequenced (×10^9^)
Tissue				
Liver	SRX4654287	Illumina	Paired	14.6
Liver	SRX3637622	Illumina	Single	2.6
Liver	SRX3441722	Illumina	Paired	12.9
WAT	SRX4003816	Illumina	Single	3.9
WAT	SRX4003814	Illumina	Single	2.3
WAT	SRX4003809	Illumina	Single	1.2
WAT	SRX4003811	Illumina	Single	2.6
BAT	SRX4003815	Illumina	Single	4.2
BAT	SRX4003813	Illumina	Single	1.6
BAT	SRX4003810	Illumina	Single	1.2
BAT	SRX4003812	Illumina	Single	1.5
Fat Diff (single cell RNA)				
Day 0	SRR1058005	Illumina	Paired	4.0
	SRR1058032	Illumina	Paired	1.8
	SRR1058033	Illumina	Paired	3.2
	SRR1058034	Illumina	Paired	3.3
Day 1	SRR1058006	Illumina	Paired	3.6
	SRR1058007	Illumina	Paired	2.4
	SRR1058008	Illumina	Paired	3.6
Day 2	SRR1058009	Illumina	Paired	2.6
	SRR1058010	Illumina	Paired	4.2
	SRR1058011	Illumina	Paired	3.2
Day 3	SRR1058012	Illumina	Paired	2.9
	SRR1058013	Illumina	Paired	3.7
	SRR1058014	Illumina	Paired	2.8
	SRR1058035	Illumina	Paired	2.1
	SRR1058036	Illumina	Paired	2.9
	SRR1058037	Illumina	Paired	3.0
Day 5	SRR1058015	Illumina	Paired	3.7
	SRR1058016	Illumina	Paired	3.4
	SRR1058017	Illumina	Paired	3.3
Day 7	SRR1058018	Illumina	Paired	2.7
	SRR1058019	Illumina	Paired	4.4
	SRR1058020	Illumina	Paired	3.6
	SRR1058038	Illumina	Paired	2.0
	SRR1058039	Illumina	Paired	3.4
	SRR1058040	Illumina	Paired	3.5
	SRR1058041	Illumina	Paired	2.9
	SRR1058042	Illumina	Paired	3.1
	SRR1058043	Illumina	Paired	3.1
	SRR1058044	Illumina	Paired	2.3
	SRR1058045	Illumina	Paired	2.9
	SRR1058046	Illumina	Paired	2.9
Day 14	SRR1058023	Illumina	Paired	2.8
	SRR1058024	Illumina	Paired	4.8
	SRR1058025	Illumina	Paired	3.7
	SRR1058026	Illumina	Paired	4.2
	SRR1058027	Illumina	Paired	3.9
	SRR1058028	Illumina	Paired	5.5
	SRR1058029	Illumina	Paired	4.0
	SRR1058030	Illumina	Paired	3.9
	SRR1058031	Illumina	Paired	4.1
Fat Diff (total RNA)				
Day 0	SRR1058047	Illumina	Paired	1.2
	SRR1058051	Illumina	Paired	1.0
Day 3	SRR1058048	Illumina	Paired	0.7
	SRR1058052	Illumina	Paired	1.0
Day 7	SRR1058049	Illumina	Paired	1.2
	SRR1058053	Illumina	Paired	1.0
Day 14	SRR1058050	Illumina	Paired	1.1
	SRR1058054	Illumina	Paired	1.0

WAT, white adipose tissue; BAT, brown adipose tissue.

### Analyzing single‐cell dynamics of *IGF1* gene expression during human adipocyte differentiation

**Figure 3:**
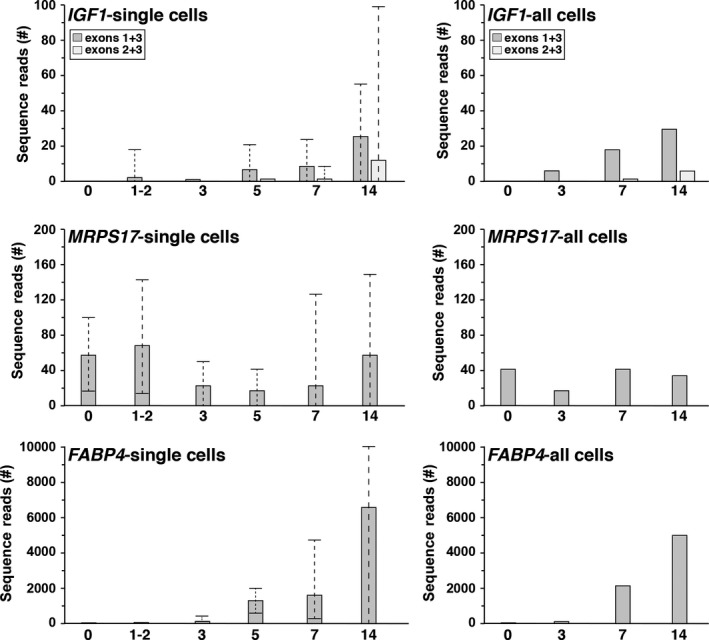
*IGF1* gene expression during human adipocyte differentiation. Left panels. Results are shown of measurements of mRNA abundance in individual human cells for *IGF1* (exons 1 + 3 = directed by *P1*; exons 2 +  3 = directed by *P2*), *FABP4*, and *MRPS17* (top to bottom panels, respectively) during adipocyte differentiation in primary culture. The graphs shown mean values plus the range of gene expression in 3–12 single cells at the following times of differentiation: day 0, days 1 and 2, day 3, day 5, day 7, and day 14. Right panels. Results of gene expression for *IGF1* (exons 1 + 3 = directed by *P1*; exons 2 + 3 = directed by *P2*), *FABP4*, and *MRPS17* (top to bottom panels, respectively) in cultured fat cells (all cells) during adipocyte differentiation at days 0, 3, 7, and 14. The mean of two experiments is shown. For both groups of studies, the gene expression libraries that were queried are listed in Table 2.

The SRA NCBI data resource also contains a series of RNA‐sequencing libraries that were prepared from single cells isolated from primary human adipocyte stem/stromal cells during their differentiation (Soumillon et al. 2014). Analysis of 42 of these libraries, representing a 14‐day time course, revealed an increase in *IGF1* gene expression directed by each promoter during adipocyte differentiation (Fig. 3), although transcript levels controlled by promoter 1 were 25‐ to 30‐fold lower at their peak on day 14 than were seen in either white or brown fat (compare with Fig. 2B). Remarkably, in different individual cells at several of the time points, levels of *IGF1* transcripts, the abundance of mRNAs for the constitutive control gene, *MRPS17*, and levels of the differentiation‐inducible gene, *FABP4*, varied over more than a 100‐fold range (Fig. 3).

### *IGF1* gene expression in rhesus macaque

**Figure 4:**
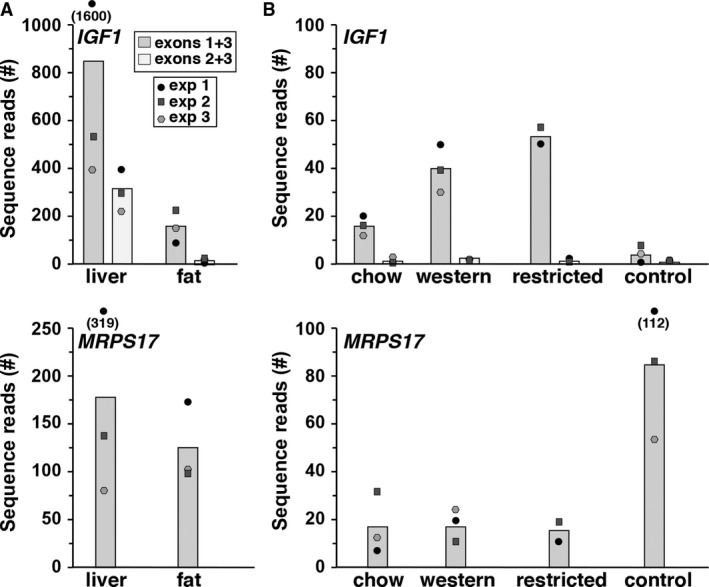
*IGF1* gene expression in macaque tissues. (A) Transcript levels for *IGF1* (exons 1 + 3 = directed by *P1*; exons 2 + 3 = directed by *P2*) and *MRPS17* (top and bottom panels, respectively) were obtained from liver and fat by interrogating the gene expression libraries listed in Table 3. Mean results of 3 experiments are shown in the bar graphs, with individual data being plotted as circles or squares. (B) Results of experiments measuring mRNA abundance for *IGF1* (exons 1 + 3 = directed by *P1*; exons 2 + 3 = directed by *P2*) and *MRPS17* (top and bottom panels, respectively) in skeletal muscle biopsies of macaques on a chow diet for 2 months, and sequentially receiving a high fat diet for 6 months followed by a calorie‐restricted chow diet for 4 months (Messaoudi et al. 2017). Also, depicted are results from control skeletal muscle RNA‐sequencing libraries. The gene expression libraries that were interrogated are listed in Table 3. Mean results of three experiments are shown in the bar graphs, with individual data being plotted as circles or squares.

Libraries were available for analysis of *IGF1* gene expression from macaque for liver and fat in the SRA NCBI (Table 3). *IGF1* mRNA was detected in both, as well as in several other organs and tissues (data not shown). Unlike what was observed in humans, steady‐state levels were eight times higher in liver than in fat for promoter 1‐derived transcripts, and were 20 times more abundant for mRNAs directed by macaque promoter 2 than for the analogous human promoter (compare Figs. 4A and 2B). Moreover, promoter 2 appeared to be ~30% as active as promoter 1 in macaque liver, versus 2.5% as active in human liver (compare Fig. 4A with Fig. 2B).

**Table 3:** Macaque RNA‐sequencing libraries screened for gene expression

	Experiment	Platform	Layout	Bases sequenced (×10^9^)
Tissue				
Liver	SRX3367372	Illumina	Paired	1.8
Liver	ERX1403306	Illumina	Single	0.9
Liver	ERX1403309	Illumina	Paired	0.8
Fat	SRX1631820	Illumina	Paired	0.5
Fat	SRX1631818	Illumina	Paired	0.5
Fat	SRX1631816	Illumina	Paired	0.5
Skeletal muscle	SRX1631821	Illumina	Paired	0.5
Skeletal muscle	SRX1631817	Illumina	Paired	0.6
Skeletal muscle	SRX1631813	Illumina	Paired	0.5
Diet and Skeletal muscle				
Chow diet	SRX2372497	Illumina	Single	3.8
	SRX2372492	Illumina	Single	3.0
	SRX2372490	Illumina	Single	3.8
Western diet	SRX2372496	Illumina	Single	3.9
	SRX2372494	Illumina	Single	3.4
	SRX2372495	Illumina	Single	4.2
Restricted diet	SRX2372493	Illumina	Single	3.7
	SRX2372491	Illumina	Single	3.7

One of the advantages of the extensive publically accessible gene expression data that now exists in SRA NCBI is that it presents opportunities for researchers to test new hypotheses by querying prior projects that may have been designed for other purposes. For example, a recent publication assessed the impact of different dietary manipulations on gene expression in skeletal muscle in male macaques (Messaoudi et al. 2017). Eight RNA‐sequencing libraries from this study have been deposited in the SRA NCBI (Table 3). These libraries were derived from serial soleus muscle biopsies from individual male macaques at the end of three dietary intervals: first, a calorie‐controlled chow diet for 2 months, then an ad libitum western diet for 6 months, and finally a chow diet for 4 months containing 70% of the calories that the animals initially ate (Messaoudi et al. 2017). Examination of the primary data from this publication revealed that *IGF1* mRNA levels showed a 2.22‐fold (=log2^1.15^) increase during caloric restriction compared with regular chow diet (Messaoudi et al. 2017).

Direct analysis of the eight RNA‐sequencing libraries from this study revealed more extensive information. Although the relative change in abundance of *IGF1* transcripts directed by promoter 1 was similar to that reported in the publication for *IGF1* mRNA levels in general, the rise in promoter‐specific gene expression was found here in both the western diet and caloric restriction groups compared with the chow diet (2.5‐fold, and 3.3‐fold, respectively, Fig. 4B), rather than just in the latter. In contrast, transcripts controlled by *IGF1* promoter 2 were minimally detected in all groups (Fig. 4B). Moreover, steady‐state levels of *IGF1* mRNA from promoter 1 from the macaques in this study were approximately three times higher in the chow diet group than in several other muscle samples found in the SRA NCBI database (Fig. 4B). Taken together, these results demonstrate the feasibility of using publically available gene expression resources to test ideas that are different from those that informed the initial project.

## Discussion

The many roles of IGF1 in growth, metabolism, and in disease pathogenesis are potentially reflected in its variable patterns of gene expression in different cell types, tissues, organs, and even species (Lowe et al. 1987; Shimatsu and Rotwein 1987; Hoyt et al. 1988; Hall et al. 1992). This study was undertaken to explore how data embedded in public databases could be used to quantify aspects of *IGF1* gene expression in mammalian species that are not otherwise tractable experimental models. The results demonstrate that the wealth of information in the SRA NCBI, which as of December 8, 2018 totals over 8.7 quadrillion searchable nucleotides from many different animal and plant species, is an outstanding resource for establishing the activity of the two *IGF1* promoters in different organs and tissues, and even for determining whether or not *IGF1* is expressed in single human adipocytes. Among the key findings here are the observations that in the organs and tissues examined, transcripts directed by *IGF1* promoter 1 are far more prevalent than those controlled by promoter 2. The only exception appears to be macaque liver, in which promoter 2 is ~30% as active as promoter 1 (Fig. 4A).

The second major result from this study was the observation that levels of a typical ‘constitutively expressed’ ‘control’ mRNA, such as *MRPS17*, also varied among different experiments and tissues (see Figs. 2B and 4). Similar results were seen in both humans and macaques for other potential controls, including transcripts for *ACTB* and *GAPDH* (data not shown). This somewhat surprising finding indicates that either these mRNAs are not optimal choices as control transcripts, or that the notion of constitutive gene expression may need to be reevaluated in the big data era.

The third observation was that gene expression in individual cells varied considerably, even under conditions in which all of the cells appeared to undergo morphological differentiation (Soumillon et al. 2014). This result was seen both in transcripts that were induced or that were thought to be relatively constant during adipocyte differentiation, and did not seem to be influenced by the complexity of the RNA‐sequencing library. The relative abundance of the different mRNAs also did not correlate with one another. Thus, the reasons for this variation do not seem to be secondary to technical issues involving library construction or DNA‐sequencing efficiency, and thus may point toward more stochastic aspects of gene regulation (Vera et al. 2016).

The fourth finding from this study relates to the potential utility of projects submitted to public gene expression databases to be repurposed by other investigators for scientific questions that are different from those posed by the original research group. As shown here, it was possible to document a promoter 1‐specific rise in *IGF1* gene expression in macaque soleus muscle during different in vivo dietary manipulations, by examining RNA‐sequencing data that had been developed for understanding general effects on dietary change in muscle (Messaoudi et al. 2017).

Finally and more broadly, the results in this paper demonstrate that there is a wealth of untapped information on gene expression in humans that now is available to investigators. The GTEx online database contains a summary of gene expression results derived by RNA sequencing from several hundred individuals, with variable numbers of samples obtained from different tissues or organs (GTEX consortium, 2015; Battle et al. 2017). As found here, *IGF1* mRNAs are detected in the majority of the 50 tissues and organs found in GTEx, with transcripts from promoter 1 predominating (Fig. 2A and data not shown). These results appear to be confirmed in data obtained from single individuals in the SRA NCBI (Fig. 2B), and lead to the general conclusion that *IGF1* mRNAs are substantially more abundant in fat than in liver in humans, in contrast to what has been found in other species (Hall et al. 1992; Gosteli‐Peter et al. 1994; Adamo 1995), including macaque (Fig. 4A). One caveat to this conclusion is that the time from tissue harvesting to RNA extraction is likely to be longer for humans than for experimental animals, and it is thus possible that transcript degradation may skew the results seen in both GTEx and the SRA NCBI RNA‐sequencing libraries.

### Final comments

One outcome of the genomics revolution is the ability to do molecular biological research with human‐derived tissues (GTEX consortium, 2015; Battle et al. 2017). When coupled to new public data resources, such as ClinGen and ClinVar (Landrum and Kattman 2018; Rivera‐Munoz et al. 2018), and more sophisticated approaches to obtain both gene expression and chromatin‐state data from single cells (Cao et al. 2018), it soon may be possible to understand how predispositions to certain traits or diseases defined by DNA polymorphisms mechanistically influence biological functions in people. As an example, recent studies indicate that alterations in DNA methylation within human *IGF1* P2 are associated with idiopathic short stature in children and with reduced responses to GH treatment (Ouni et al. 2016a,b), although the genetic underpinnings of these epigenetic modifications are not yet known. The ability to tap large‐scale genomic and genetic resources to develop and validate new ideas clearly complements smaller scale gene expression experiments, using RT‐RCR or even RNA‐sequencing strategies. It also should further extend opportunities to maximize genome‐scale observations for disease prevention and more broadly for improving human health and health span (Berryman et al. 2008; Gallagher and LeRoith 2011; Pollak 2012; Gems and Partridge 2013).

## Conflict of Interest

None declared.
